# YAP/TAZ Signaling in the Pathobiology of Pulmonary Fibrosis

**DOI:** 10.3390/cells13181519

**Published:** 2024-09-10

**Authors:** Kostas A. Papavassiliou, Amalia A. Sofianidi, Fotios G. Spiliopoulos, Vassiliki A. Gogou, Antonios N. Gargalionis, Athanasios G. Papavassiliou

**Affiliations:** 1 First University Department of Respiratory Medicine, Medical School, ‘Sotiria’ Hospital, National and Kapodistrian University of Athens, 11527 Athens, Greece; konpapav@med.uoa.gr (K.A.P.); gogvanessa@gmail.com (V.A.G.); 2 Department of Biological Chemistry, Medical School, National and Kapodistrian University of Athens, 11527 Athens, Greece; amsof.00@gmail.com (A.A.S.); spiliopoulosfotis99@gmail.com (F.G.S.); 3 Laboratory of Clinical Biochemistry, Medical School, ‘Attikon’ University General Hospital, National and Kapodistrian University of Athens, 12462 Athens, Greece; agargal@med.uoa.gr

**Keywords:** pulmonary fibrosis, idiopathic pulmonary fibrosis, Hippo pathway, YAP, TAZ

## Abstract

Pulmonary fibrosis (PF) is a severe, irreversible lung disease characterized by progressive scarring, with idiopathic pulmonary fibrosis (IPF) being the most prevalent form. IPF’s pathogenesis involves repetitive lung epithelial injury leading to fibroblast activation and excessive extracellular matrix (ECM) deposition. The prognosis for IPF is poor, with limited therapeutic options like nintedanib and pirfenidone offering only modest benefits. Emerging research highlights the dysregulation of the yes-associated protein (YAP)/transcriptional coactivator with PDZ-binding motif (TAZ) signaling pathway as a critical factor in PF. YAP and TAZ, components of the Hippo pathway, play significant roles in cell proliferation, differentiation, and fibrosis by modulating gene expression through interactions with TEA domain (TEAD) transcription factors. The aberrant activation of YAP/TAZ in lung tissue promotes fibroblast activation and ECM accumulation. Targeting the YAP/TAZ pathway offers a promising therapeutic avenue. Preclinical studies have identified potential treatments, such as trigonelline, dopamine receptor D1 (DRD1) agonists, and statins, which inhibit YAP/TAZ activity and demonstrate antifibrotic effects. These findings underscore the importance of YAP/TAZ in PF pathogenesis and the potential of novel therapies aimed at this pathway, suggesting a new direction for improving IPF treatment outcomes. Further research is needed to validate these approaches and translate them into clinical practice.

## 1. Introduction

Pulmonary fibrosis (PF) is a rare interstitial lung disease that causes progressive and irreversible lung scarring [1]. It is divided based on its etiology into idiopathic pulmonary fibrosis (IPF), when the exact cause of PF is unknown, and secondary pulmonary fibrosis (SPF), with the latter mainly attributed to rheumatic diseases (e.g., rheumatoid arthritis, systemic sclerosis, and lupus), infections, long term exposure to certain toxins (e.g., asbestos, silica dust, coal dust, and certain metals), radiation therapy for malignancies (particularly in the chest area), and drugs, including chemotherapy agents (e.g., bleomycin), antiarrhythmics (e.g., amiodarone), and antibiotics (e.g., nitrofurantoin) [2]. Despite our lack of knowledge regarding the pathophysiology of IPF, data suggest that this disease is a result of continuous injury to the lung epithelium. In response to lung injury, the normal repair mechanisms become dysregulated, and as a result, instead of proper healing, abnormal wound healing is characterized by excessive activation of fibroblasts and their differentiation into myofibroblasts. Activated fibroblasts and myofibroblasts secrete large amounts of extracellular matrix (ECM) components, particularly collagen. The excessive deposition of ECM leads to the thickening and stiffening of lung tissue, reducing its elasticity and function [3]. IPF is an extremely fatal lung disease with an extremely poor prognosis, similar to aggressive lung malignancies [4]. Notably, localized non-small cell lung cancer (NSCLC) has a 5-year survival rate of 65%, according to the American Cancer Society [5], while IPF has a 5-year survival rate of approximately 54% [6] and a median survival of only 2–5 years after diagnosis [7,8].

The high mortality rate of PF is strongly associated with the limited therapeutic options available to treat this disease. Pharmacological agents used in the management of IPF patients do not treat the disease but rather decelerate its progression and include nintedanib and pirfenidone [9]. Nintedanib functions as an inhibitor of tyrosine kinases with antifibrotic, anti-inflammatory, and vascular remodeling effects [10]. Pirfenidone is a broad-spectrum antifibrotic drug that inhibits the proliferation of fibroblasts, the production of collagen, and the differentiation of myofibroblasts [11]. However, both nintedanib and pirfenidone have side effects that vary in their impact on different patients, presenting with varying degrees of severity; nintedanib is closely linked to diarrhea and other gastrointestinal symptoms, while pirfenidone is more likely to cause adverse skin reactions, especially photosensitivity, as well as gastrointestinal intolerance, primarily in the form of nausea [12,13,14]. This variability in adverse effects complicates treatment for medical practitioners. So far, the only effective therapy for IPF patients that decreases mortality is lung transplantation [15]. However, lung transplants are not without complications, which further highlights the need for novel therapeutic approaches for PF. Re-examining the molecular landscape behind PF pathophysiology revealed the dysfunction of yes-associated protein (YAP)/transcriptional coactivator with PDZ-binding motif (TAZ) signaling as a major cause of PF. The YAP/TAZ cascade has been identified as a critical regulator of fibroblast activation and ECM synthesis in PF. Activation of the Hippo pathway components YAP and TAZ in alveolar epithelial cells and fibroblasts contributes to lung regeneration and harmful remodeling after injury, with dysregulation of this pathway implicated in the progression of lung fibrosis [16]. The aim of this review is to delve into the role of the YAP/TAZ cascade in the pathogenesis and progression of PF. Therapeutic efforts towards targeting several pathways associated with YAP/TAZ will be reviewed as well.

## 2. YAP/TAZ–TEAD Biology and Signaling Mechanism

The Hippo cascade, initially identified in the fruit fly [17], is an evolutionary preserved signaling network consisting of two main components that serve as transcriptional cofactors, namely, YAP and TAZ [18,19]. The YAP protein is encoded by the YAP1 gene, which is located on human chromosome 11q22. This protein consists of several functional domains, including tea domain (TEAD)-binding (TB) region, two WW domains, an SH3-binding motif, a coiled-coil domain, a transcription activation domain, an N-terminal proline-rich domain, and a C-terminal PDZ-binding motif [20]. The TAZ protein, encoded by the *TAZ* gene on human chromosome 3q23-q24, differs in terms of structure from YAP by exhibiting only a single WW domain and no SH3-binding motif or proline-rich domain [20]. Both proteins shuttle between the nucleus and cytosol [21]. They interact with the PPXY (P: Proline, X: any amino acid, Y: Tyrosine) motifs of various other transcription factors via their WW domain, while their TB region attaches to proteins with a DNA-binding domain, such as the TEAD 1–4 transcription factors, inducing gene expression [22].

The core component of the Hippo pathway consists of two regulatory kinases, STE20-like protein kinase 1/2 (MST1/2) and large tumor suppressor 1/2 (LATS1/2) [18]. The aforementioned kinases phosphorylate the YAP/TAZ complex. Upstream physiological or pathological signals trigger the formation of MST1/2-Salvador 1 (SAV1) complexes, leading to the phosphorylation of LATS1/2 and MOB kinase activator 1 (MOB1). In turn, these activated proteins go on to phosphorylate the Hippo transcriptional effectors YAP/TAZ [20,23]. Finally, the phosphorylated YAP/TAZ complex associates with 14–3–3 proteins, resulting in YAP/TAZ cytoplasmic retention and degradation. When Hippo signaling is turned off, YAP/TAZ undergo nuclear translocation, where they interact with TEAD 1-4 transcription factors and regulate target gene expression [18,20]. The activity status of Hippo signaling is affected by multiple factors, such as the upstream modulator neurofibromatosis type 2 (NF2), G protein-coupled receptors (GPCRs), which either activate or deactivate the YAP/TAZ complex and mechanotransduction (signaling induced by extracellular/intracellular mechanical cues) [20].

The Hippo pathway regulates physiological processes, including the proliferation and differentiation of cells, the development of organs, embryogenesis, and tissue regeneration/wound healing [24]. When dysfunctional, it is linked to various cancer types, such as mesothelioma [20], breast [25], and liver cancer [26], alongside various diseases that affect multiple organs, such as retinal-related diseases [27], cardiac diseases [28], pulmonary diseases [29], renal diseases [30], central nervous system disorders [31], and immune dysfunction [24,32]. Its most recent implication has been in fibrosis-related diseases [16], with PF being the main objective of our review.

## 3. YAP/TAZ Signaling Pathway in PF

Under normal conditions, YAP and TAZ are implicated in the development of the lungs and their physiology [33]. On the other hand, aberrant YAP/TAZ signaling participates in the pathophysiology of chronic respiratory diseases, such as lung malignancies, pulmonary hypertension, chronic obstructive pulmonary disease (COPD), asthma, lung infections, and PF [34]. The hallmark of PF is the accumulation of extracellular matrix (ECM) and collagen within the lung’s interstitial space [35]. YAP and TAZ were first identified as primary regulators of fibroblast activation and matrix synthesis in PF in 2015 [36]. They were found to be highly expressed in fibrotic lung tissue, but not in healthy lung tissue, with TAZ showing prominent nuclear expression in spindle-shaped fibroblastic cells [36]; nuclear translocation of YAP/TAZ is the key determinant for their activation [37]. Liu et al. demonstrated enhanced localization of TAZ in IPF fibroblast nuclei, while in normal tissue, YAP/TAZ showed mainly cytoplasmic localization [36]. It was also found that siRNA knockdown and the subsequent loss of function of YAP and TAZ reduces key fibrogenic activities in IPF fibroblasts, while the gain of function of YAP or TAZ is sufficient to promote fibrogenesis both in vitro and in vivo [36]. Specifically, fibroblast activation is regulated by YAP/TAZ in response to matrix stiffness by upregulating SERPINE1, encoding plasminogen activator inhibitor 1 (PAI-1) [36]. PAI-1 is highly profibrotic; excessive PAI-1 contributes to abnormal accumulation of collagen and ECM [38]. TGF-β signaling augments the expression of PAI-1 [39], but Liu et al. highlight that the matrix can maintain a profibrotic state independently of this key fibrogenic cytokine [36]. Notably, knocking down the expression of YAP/TAZ downregulated PAI-1 expression induced by TGF-β [36]. Similarly, in 2022, Link et al. demonstrated that TGF-β-related activation of lung fibroblasts was blocked by YAP/TAZ knockdown [40], suggesting that the YAP/TAZ cascade is strongly involved in the TGF-β signaling pathway. The same study showed that YAP/TAZ knockdown in IPF human lung fibroblasts led to the decreased expression of Acta2, Cnn1, and Tagln, three profibrotic contractile genes [40].

Zmajkovicova et al. further investigated the role of YAP/TAZ in the TGF-β cascade. GPCRs are known regulators of the YAP/TAZ-TEAD signaling axis [41]. GPCR ligands, such as lysophosphatidic acid (LPA), sphingosine-1-phosphate (S1P), and thrombin, synergize with TGF-β1 in human fibroblasts derived from the dermis, promoting the production of extracellular matrix, myofibroblast differentiation biomarkers, and cytokine secretion. TGF-β1 activates Smad2/3/4 complexes, leading to their transport into the nucleus and subsequent binding to YAP, which ultimately drives the upregulation of target gene expression associated with fibrinogenesis. GPCR ligand binding to GPCRs trigger initiates Rho activation, which dephosphorylates YAP and facilitates its accumulation in the nucleus. This process enhances YAP/Smad-associated target gene transcription. Conversely, agents such as latrunculin B, forskolin, Rho inhibitors, 2-deoxy-D-glucose (2-DG), or GPCR ligands that increase the intracellular concentration of cAMP result in low levels of YAP within the nucleus, thereby reducing the TGF-β1-responsive gene transcription induced by YAP/Smad that promotes fibrinogenesis [42]. Interestingly, S1P levels are increased in IPF [43].

The sphingosine kinase 1 (SPHK1)/S1P pathway represents an important contributor to IPF development [44]. SPHK1 displays increased expression in IPF human lung tissue and IPF mice models [45]. Huang et al. demonstrated that SPHK1 knockout in fibroblasts reduces bleomycin- and TGF-β-induced YAP1 expression. These findings hint at a potential crosstalk between SPHK1/S1P and Hippo signaling in TGF-mediated fibroblast differentiation, as well as fibrosis. Blocking SPHK1 activity was found to attenuate BLM- or TGF-β-related mitochondrial ROS generation; mitochondrial ROS is necessary for TGF-induced fibronectin and alpha-smooth muscle actin (a-SMA) expression in lung fibroblasts [44].

Tank-binding protein kinase-1 (TBK1) has also been demonstrated to control TGF-mediated lung fibroblast activation by affecting the stability of YAP/TAZ [46]. TBK1 is a serine/threonine protein kinase that interacts with various substrates, including those involved in the immune response, inflammation, autophagy, cell proliferation and growth, and insulin signaling [47]. TBK1 emerged as crucial for fibroblast activation, and it plays a vital role in maintaining YAP/TAZ protein stability and nuclear localization. While TBK1 is crucial for TGF-β-mediated fibroblast activation, it does not directly respond to TGF-β. Instead, TBK1 expression and activation are influenced by mechanotransduction and physical cues such as matrix stiffness and cell density [46].

Furthermore, it is well established that the YAP and TEAD complex translocates into the nucleus, where it interacts with transcription factors and other DNA-binding proteins to regulate target gene transcription [48]. Chen et al. found that the profibrotic effect of YAP was facilitated by upregulating the transcription of Twist1 through binding to TEAD [49]. Twist1, a basic helix–loop–helix transcription factor, is crucial in epithelial-mesenchymal transition (EMT) and plays a significant role in various fibrotic diseases [50]. Tan et al. also investigated the role of Twist1 in PF. In this study, the loss of Twist1 in collagen-producing cells resulted in increased BLM-induced PF, driven by elevated levels of the T cell chemoattractant CXCL12. Additionally, Twist1 expression was found to be linked to T cell dysregulation in IPF patients [51]. Thus, while Twist plays multiple roles in the progression of IPF, its molecular effects require further investigation.

**Figure 1:**
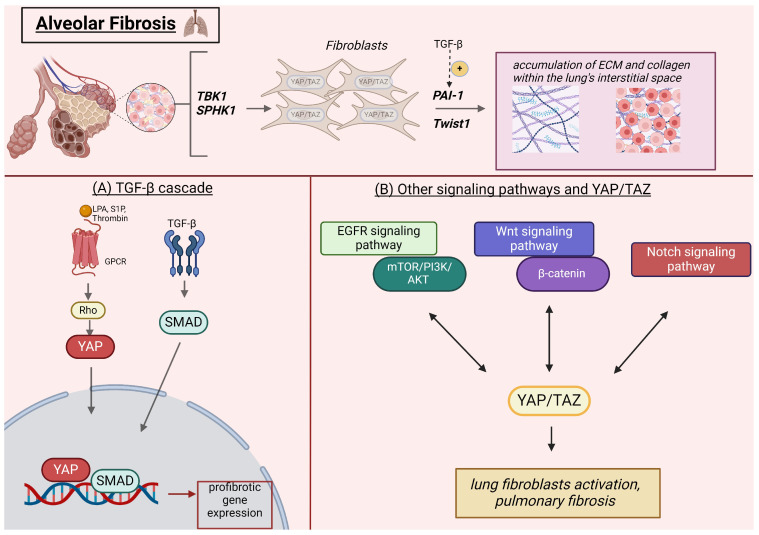
YAP/TAZ signaling pathway in the pathogenesis of PF. YAP and TAZ are highly expressed in fibrotic lung tissue, with TAZ showing prominent nuclear expression in spindle-shaped fibroblastic cells. At a molecular level, TBK1 and SPHK1 promote the fibrotic effects of YAP/TAZ, which are regulated through interactions with PAI-1 and Twist1. TGF-β signaling augments the expression of PAI-1. (**A**) The role of YAP/TAZ in the TGF-β cascade. GPCR ligands, such as LPA, S1P, and thrombin, facilitate the accumulation of YAP in the nucleus through the mediation of Rho. TGF-β activates Smad2/3/4 complexes, leading to their translocation into the nucleus, where they utilize YAP as a coactivator to drive the transcription of fibrogenic YAP/Smad target genes. (**B**) In the pathogenesis of IPF, YAP/TAZ interacts with several other signaling pathways, such as EGFR, Wnt, and Notch, to promote lung fibroblast activation and pulmonary fibrosis. This figure was created based on the tools provided by Biorender.com (https://biorender.com/; accessed 5 August 2024). EGFR: epidermal growth factor receptor, GPCR: G protein-coupled receptor, IPF: idiopathic pulmonary fibrosis, LPA: lysophosphatidic acid, PAI-1: plasminogen activator inhibitor-1, Rho: ras homolog family member, S1P: sphingosine-1-phosphate, Smad: mothers against decapentaplegic homolog, SPHK1: sphingosine kinase 1, TAZ: transcriptional coactivator with PDZ-binding motif, TBK1: TANK binding kinase 1, TGF-β: transforming growth factor beta, Twist1: Twist Family BHLH transcription factor 1, Wnt: wingless-related integration site, YAP: yes-associated protein.

During IPF, YAP/TAZ interacts with several other signaling pathways [52]. The EGFR signaling pathway regulates nuclear YAP/TAZ by activating the Ras/Raf/MEK/ERK and PI3K/Akt/mTORC1 signaling axes [52]. In the pathogenesis of IPF, YAP engages with the mTOR/PI3K/AKT signaling pathway to control the proliferation, migration, differentiation, and polarity of lung epithelial cells [53]. Overall, the activation of these signal transduction axes has been linked to poor prognosis of patients with PF [52,54]. Wnt/β-catenin signaling is another key player involved in the pathogenesis of PF. Sun et al. demonstrated that its activation modulates mesenchymal stem cell differentiation and is associated with lung fibroblast activation, PF, and tissue repair processes both in vitro and in vivo [55]. YAP/TAZ and Wnt signaling pathways are strongly connected to one another; the Wnt pathway affects PF development via β-catenin, whereas YAP/TAZ affect β-catenin expression. In the absence of Wnt ligands, YAP/TAZ degrade β-catenin through a destruction complex, while in the presence of Wnt ligands, YAP/TAZ release from the destruction complex, and β-catenin undergoes nuclear translocation, binds to its binding partner T-cell factor (TCF), and enables gene transcription [52]. Wnt signaling can reciprocally enhance TGF-β activity, leading to sustained fibroblast activation and ECM deposition [56]. The Notch signaling pathway is also crucial in various fibrotic diseases, including PF [57]. The activated YAP/TAZ complex is shuttled within the nucleus, triggering Notch receptor and/or Notch ligand expression [52]. Furthermore, simultaneous YAP/TAZ and Notch signaling activation promotes the entry of both YAP/TAZ and the transcriptionally active Notch intracellular domain (NICD) peptide in the nucleus. This cooperative interaction regulates the transcription of common target genes [52]. The involvement of the YAP/TAZ signaling pathway in the pathobiology of PF is illustrated in Figure 1.

**Figure 2:**
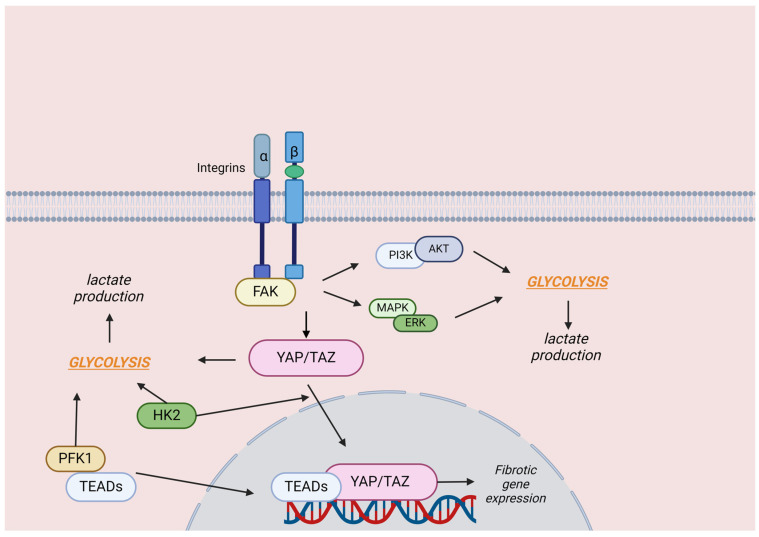
The role of YAP/TAZ in regulating glycolytic reprogramming in lung fibrosis. YAP/TAZ enhances the expression of glycolytic enzymes, boosting glucose metabolism and lactate production. Integrins, through signaling pathways like PI3K/Akt and MAPK/ERK, further regulate glycolysis, impacting glucose utilization and energy metabolism. Integrins/FAK regulate glycolysis via the YAP/TAZ axis. This axis facilitates the nuclear translocation of YAP/TAZ, enhancing their fibrotic effects. Additionally, HK2, a key glycolytic enzyme, is crucial for YAP/TAZ nuclear translocation. The activation of lung fibroblasts is often marked by upregulation of glycolytic enzymes, such as PFK1, which interacts with YAP/TAZ coactivator TEADs, promoting fibroblast differentiation. This figure was created based on the tools provided by Biorender.com (https://biorender.com/; accessed 5 August 2024). Akt: protein kinase B, ERK: extracellular signal-regulated kinase, FAK: focal adhesion kinase, HK2: hexokinase 2, MAPK: mitogen-activated protein kinase, PI3K: phosphoinositide 3-kinase, PFK1: phosphofructokinase-1, TEADs: TEA domain transcription factors, YAP: yes-associated protein, TAZ: transcriptional coactivator with PDZ-binding motif.

Recently, Yan et al. explored the effect of YAP/TAZ signaling in glycolytic reprogramming in the setting of lung fibrosis [58]. Evidence indicates that IPF patients show metabolic dysregulation of glucose, lipids, hormones, and other metabolites in their lungs [58,59,60]. This dysregulation causes abnormal metabolic product levels, interfering with the homeostatic processes that maintain the pulmonary microenvironment in balance [58,61]. For example, lactate, a glycolytic byproduct, is found in abundance in human IPF lung tissue [58]. Research has demonstrated that glycolysis is enhanced in fibrotic regions of IPF lungs and may be used as a predictive biomarker with respect to lung function decline and mortality [62]. Although data regarding the association of YAP/TAZ with glycolysis is scarce, available evidence indicates that key enzymes involved in glycolysis are upregulated by YAP/TAZ, thus increasing glucose metabolism and intracellular production of lactate [58,63,64]. Furthermore, a study suggests that upstream signaling via integrins/FAK activates the YAP/TAZ pathway, which leads to the regulation of glycolysis [58]. Enhanced glycolysis seems to enable YAP/TAZ entry into the nucleus and induction of gene expression promoting fibrosis. Moreover, in order for YAP/TAZ to enter the nucleus, the presence of the key glycolytic enzyme HK2 is required [65]. Phosphofructokinase-1 (PFK1) is another glycolytic enzyme that appears to be upregulated in activated human lung fibroblasts. Additionally, PFK1 has been shown to bind to TEADs, supporting their interaction with YAP/TAZ, which may explain how this glycolytic enzyme achieves to regulate the differentiation of fibroblasts [66]. Overall, these findings suggest that YAP/TAZ signaling promotes PF progression via reprogramming glycolytic pathways (Figure 2).

## 4. Targeting the YAP/TAZ Signaling Cascade in PF

Due to the limited therapeutic options and the poor survival rate associated with PF, novel treatments are being actively explored. The YAP/TAZ signaling pathway has been implicated in the pathogenesis of PF, redirecting the focus of therapeutic development towards this pathway. However, current therapeutic approaches targeting the YAP/TAZ signaling pathway are still in the preclinical evaluation stage.

The first molecule targeting the YAP/TAZ signaling pathway to undergo preclinical evaluation for the treatment of PF is trigonelline. Trigonelline, a natural alkaloid plant with diverse pharmacological actions [67], is present in fenugreek seeds [68] and has also been found in coffee and clover [69,70]. Zeyada et al. investigated the role of trigonelline in pulmonary inflammation and fibrosis in vivo [71]. Using bleomycin to induce fibrosis in rats, they observed amplification of the SPHK1/S1P/Hippo signaling pathway and its fibrogenic target genes. Subsequently, both prophylactic and therapeutic doses of trigonelline and pirfenidone were administered to the test subjects. This combinatorial treatment significantly decreased SPHK1 transcription in the lung, S1P protein levels, YAP-1 protein expression and nuclear translocation, and TAZ transcription. Furthermore, trigonelline and pirferidone reduced the expression of the YAP/TAZ signaling profibrotic genes assessed in the current study, namely LOX, PAI-1, CYR61, and CTGF genes [71].

Another preclinical therapeutic approach for inhibiting the YAP/TAZ pathway in lung fibroblasts involves targeting the dopamine receptor D1 (DRD1) [72]. Haak et al. demonstrated that the Gαs-coupled DRD1 is preferentially expressed in lung and liver mesenchymal cells. DRD1 agonists selectively inhibited YAP/TAZ function in these cells, shifting their environment from profibrotic to fibrosis-resolving. This resulted in the reversal of extracellular matrix deposition and stiffening in vitro, as well as tissue fibrosis in vivo in mouse models [72]. Among the agonists tested, dihydrexidine (DHX) was the most effective, leading to elevated cAMP levels, YAP phosphorylation, and YAP/TAZ cytoplasmic retention or degradation [72]. Moreover, the YAP/TAZ pathway is known to be essential for organ and tissue regeneration and angiogenesis [73,74]. This cell-selective approach proposed by Haak et al. preserves YAP/TAZ function in epithelial and endothelial compartments, which is important for maintaining the capability of injury resolution and tissue repair [72]. The significance of the dopamine pathway in PF is further underscored by the finding that aromatic L-amino acid decarboxylase (DOPA decarboxylase, DDC), the enzyme crucial for the final step in dopamine biosynthesis, is reduced in the lungs of individuals with IPF. This enzyme’s expression inversely correlates with disease severity, suggesting a protective role for dopamine signaling that is compromised in PF [72].

In another study, Santos et al. used high-throughput small-molecule screening on primary human lung fibroblasts and identified that hydroxymethylglutaryl-coenzyme A (HMG-CoA) reductase inhibitors, commonly known as statins, can target YAP [75]. Statins have been found to promote YAP phosphorylation, leading to its retention in the cytoplasm and subsequent degradation. This process ultimately results in the exclusion of YAP from the nucleus and its subsequent inactivation in human lung fibroblasts [75]. Santos et al. tested the effects of simvastatin in vivo using bleomycin-induced IPF mouse models. Notably, simvastatin was found to modulate YAP localization in vivo and attenuate established lung fibrosis in bleomycin-challenged mouse models of IPF [75]. This discovery validated statins as effective antifibrotic agents in the lung, prompting further studies to explore their clinical potential in IPF and other fibrotic diseases.

Another study by Zhao et al. highlighted the therapeutic effects of melatonin, a neurohormone, in PF [76]. By activating YAP/TAZ signaling, melatonin was able to mitigate TGF-β-induced fibrogenesis in lung fibroblasts, promoting YAP nuclear entry and increasing its cytoplasmic inactivation and degradation. Additionally, melatonin was found to alleviate PF in mice induced with bleomycin [76]. However, luzindole, which blocks melatonin receptors, diminished the beneficial effects of melatonin, indicating that the latter hinders PF through a receptor-dependent mechanism [76]. The in vitro and in vivo findings by Zhao et al. suggest that melatonin administration may represent a potential new strategy to improve lung fibrosis outcomes.

In an effort to expand our arsenal of therapeutic strategies targeting the YAP/TAZ pathway, herbal compound prescriptions have consistently proven to be valuable and effective tools for discovering numerous therapeutic agents [77]. Icariin is the primary active ingredient derived from the medicinal plant *Epimedium brevicornum* Maxim, known for its beneficial effects on various diseases, including bone loss, cancer, cardiovascular disease, neurodegenerative disorders, and nonalcoholic steatohepatitis (NASH) [78]. It has also been reported to have anti-inflammatory, antioxidant, anti-angiogenic, and antifibrotic effects [79,80]. Li et al. found that icariin reduces the accumulation of type IV collagen and fibronectin in mesangial cells exposed to high glucose levels. This effect is achieved through the inhibition of TGF-β production via the G protein-coupled estrogen receptor 1 [81]. Moreover, Sun et al. demonstrated that icariin may effectively prevent, alleviate, or reduce inflammatory responses and fibrotic diseases in various organs and tissues. Icariin was found to reduce leukocyte influx in bronchoalveolar lavage fluid and diminish pulmonary inflammation induced by bleomycin [82]. Notably, the antifibrotic effect of icariin seems to be mediated by its inhibition of YAP, the key transcriptional regulator of the Hippo pathway [83]. In summary, icariin may be a promising drug candidate for preventing and treating PF due to its inhibition of the Hippo signaling pathway.

Finally, Zmajkovicova et al. identified a novel mechanism of YAP/TAZ inhibition through prostacyclin receptor activation, which suppresses profibrotic (myo)fibroblast activity [84]. They explored the antifibrotic effects of the selective prostacyclin receptor agonist ACT-333679 (Selexipag) in primary human lung fibroblasts. ACT-333679 was found to inhibit the TGF-β1-induced transformation of fibroblasts into myofibroblasts, as well as their proliferation, ECM synthesis, and the secretion of IL-6 and PAI-1. Additionally, it exhibited relaxant effects in cell contraction assays and was capable of reversing an established myofibroblast phenotype. Crucially, ACT-333679 elevated cAMP levels, resulting in YAP/TAZ nuclear exclusion and subsequent suppression of YAP/TAZ-dependent profibrotic gene transcription [84]. This discovery paves the way for the development of prostacyclin receptor agonists as a potential treatment for IPF.

Table 1 presents the molecules developed to date targeting the YAP/TAZ signaling cascade in PF, which are undergoing preclinical evaluation.

## 5. Future Perspectives—Outlook

The journey from the start of a discovery program to the point where national drug regulatory agencies grant marketing approval typically spans 12 to 15 years [85]. This means that we have a long road ahead for the approval of PF drugs targeting the YAP/TAZ cascade. All molecules developed to target the Hippo pathway are still in the preclinical stage of evaluation. Similarly, this is the case for molecules targeting fibrosis in other organs, such as the heart, liver, kidneys, and skin [16]. However, when considering lung diseases, several agents targeting the YAP/TAZ cascade in pleural mesothelioma, a malignant lung disease, are currently being evaluated in phase 1 studies. These include agents targeting YAP/TAZ expression, TEAD palmitoylation, or the interaction between YAP/TAZ and TEAD [20], offering a promising example of novel therapeutic approaches for PF. Combining YAP/TAZ inhibitors with other antifibrotic therapies, such as pirfenidone and nintedanib, may also enhance therapeutic efficacy and overcome resistance mechanisms. Notably, recent studies have demonstrated that nintedanib exerts antifibrotic effects by impairing TBK1-mediated YAP/TAZ nuclear translocation [86], thereby targeting the YAP/TAZ pathway. Combining nintedanib with a YAP/TAZ inhibitor that targets another part of this cascade could be a promising approach for treating PF.

Most recently, Graham et al. explored an indirect method of targeting the YAP/TAZ cascade [87]. The conduction of a phenotypic high-throughput screen led to the identification of BAY-856, a potent indirect YAP/TAZ inhibitor. Further probing revealed that BAY-856 and similar inhibitors directly target the geranylgeranyltransferase-I (GGTase-I) complex. These pharmacological agents bind to and block Rho-GTPases, resulting in YAP/TAZ inactivation and cancer cell proliferation inhibition [87]. While Graham et al. focused their studies on cancer cells, the involvement of the Hippo pathway in the pathobiology of PF suggests that these findings could be applicable to lung fibrosis as well.

The interaction of YAP/TAZ with other signaling pathways, such as TGF-β, Wnt, and Notch, adds layers of complexity that are not fully understood. Research should explore the exact mechanisms of these interactions and how inhibiting YAP/TAZ impacts these other pathways. This could uncover new therapeutic strategies or reveal challenges in targeting multiple pathways simultaneously. Huang et al. most recently demonstrated that ShaShen-MaiDong decoction (SMT), a traditional Chinese medicine, could enrich the therapeutic arsenal for PF by simultaneously modulating the MAPK, TGF-β/Smad, and YAP/TAZ signaling pathways [88].

While the focus of current research largely revolves around treating PF after diagnosis, prevention strategies are equally crucial in reducing the incidence and severity of the disease. Early detection of risk factors, lifestyle modifications, and interventions targeting key molecular pathways, such as the YAP/TAZ signaling axis, may play a vital role in slowing or halting the progression of PF before it reaches an advanced stage. Building on this concept, Ohto-Fujita et al. highlighted the potential of eggshell membranes, along with their key components, lysozyme and ovotransferrin, as a promising preventive approach for PF. They demonstrated that these substances could stimulate the secretion of decorin, an endogenous antifibrotic mediator, from lung fibroblasts, which interferes with the Hippo cascade, offering a novel strategy to combat the development of fibrosis [89].

## 6. Conclusions

PF, a rare and progressive interstitial lung disease, presents significant clinical challenges due to its poor prognosis and limited therapeutic options. Central to its pathophysiology is the dysregulation of the YAP/TAZ signaling pathway, which influences fibroblast activation and ECM deposition. The interaction of YAP/TAZ with other pathways, such as TGF-β, EGFR, Wnt, and Notch, further complicates the disease mechanism. Recent research has unveiled potential therapeutic agents targeting the YAP/TAZ axis, including natural compounds like trigonelline, dopamine receptor agonists, and statins, all showing promise in preclinical models. These advancements highlight a crucial shift towards targeting molecular pathways to develop effective PF treatments. Understanding the intricate roles of YAP/TAZ in glycolytic reprogramming and fibroblast differentiation opens new avenues for intervention. However, translating these findings from bench to bedside remains a significant hurdle. Continued exploration and clinical trials are imperative to establish these novel therapies, offering hope for improved outcomes in PF patients.

## Figures and Tables

**Table 1:** Preclinical targeting of the YAP/TAZ signaling cascade in PF.

Compound	Mechanism of Action	Experimental Study
Trigonelline	Attenuates lung SPHK1/S1P axis, decreases YAP/TAZ profibrotic phenotypes, and promotes matrix degradation	Mitigates BML-induced PF in rats [71]
Dihydrexidine	DRD1 agonist elevates cAMP and promotes YAP/TAZ Ser127 phosphorylation	Reverses fibrosis in mice [72]
Simvastatin	Modulates YAP/TAZ localization	Attenuates established lung fibrosis in BML-induced PF in mice [75]
Melatonin	Promotes the translocation of YAP/TAZ into the nucleus	Mitigates BML-induced PF in mice, Attenuates TGF-β-induced fibrogenesis by activating the YAP/TAZ pathway [76]
Icariin	Blocks YAP function	Prevents BML-induced PF in rats [83]
ACT-333679 (Selexipag)	YAP/TAZ inhibition through prostacyclin receptor activation	Inhibits the TGF-β1-induced transformation of fibroblasts into myofibroblasts [84]

BML, bleomycin; DRD1, dopamine receptor D1; PF, pulmonary fibrosis.

## Data Availability

Not applicable.

## References

[1] Wuyts W.A., Agostini C., Antoniou K.M., Bouros D., Chambers R.C., Cottin V., Egan J.J., Lambrecht B.N., Lories R., Parfrey H. (2013). The Pathogenesis of Pulmonary Fibrosis: A Moving Target. Eur. Respir. J..

[2] Wilson M.S., Wynn T.A. (2009). Pulmonary Fibrosis: Pathogenesis, Etiology and Regulation. Mucosal Immunol..

[3] Mei Q., Liu Z., Zuo H., Yang Z., Qu J. (2022). Idiopathic Pulmonary Fibrosis: An Update on Pathogenesis. Front. Pharmacol..

[4] Fujimoto H., Kobayashi T., Azuma A. (2015). Idiopathic Pulmonary Fibrosis: Treatment and Prognosis. Clin. Med. Insights Circ. Respir. Pulm. Med..

[5] American Cancer Society (2024). Lung Cancer Survival Rates. https://www.cancer.org/cancer/types/lung-cancer/detection-diagnosis-staging/survival-rates.html.

[6] Tsubouchi K., Hamada N., Tokunaga S., Ichiki K., Takata S., Ishii H., Kitasato Y., Okamoto M., Kawakami S., Yatera K. (2023). Survival and Acute Exacerbation for Patients with Idiopathic Pulmonary Fibrosis (IPF) or Non-IPF Idiopathic Interstitial Pneumonias: 5-Year Follow-up Analysis of a Prospective Multi-Institutional Patient Registry. BMJ Open Respir. Res..

[7] Nathan S.D., Shlobin O.A., Weir N., Ahmad S., Kaldjob J.M., Battle E., Sheridan M.J., du Bois R.M. (2011). Long-Term Course and Prognosis of Idiopathic Pulmonary Fibrosis in the New Millennium. Chest.

[8] Ley B., Ryerson C.J., Vittinghoff E., Ryu J.H., Tomassetti S., Lee J.S., Poletti V., Buccioli M., Elicker B.M., Jones K.D. (2012). A Multidimensional Index and Staging System for Idiopathic Pulmonary Fibrosis. Ann. Intern. Med..

[9] Wilson K.C., Raghu G. (2015). The 2015 Guidelines for Idiopathic Pulmonary Fibrosis: An Important Chapter in the Evolution of the Management of Patients with IPF. Eur. Respir. J..

[10] Van Den Hoogen L.L., Van Laar J.M. (2020). Targeted Therapies in Systemic Sclerosis, Myositis, Antiphospholipid Syndrome, and Sjögren’s Syndrome. Best Pract. Res. Clin. Rheumatol..

[11] Aimo A., Spitaleri G., Nieri D., Tavanti L.M., Meschi C., Panichella G., Lupón J., Pistelli F., Carrozzi L., Bayes-Genis A. (2022). Pirfenidone for Idiopathic Pulmonary Fibrosis and Beyond. Card. Fail. Rev..

[12] Proesmans V.L.J., Drent M., Elfferich M.D.P., Wijnen P.A.H.M., Jessurun N.T., Bast A. (2019). Self-Reported Gastrointestinal Side Effects of Antifibrotic Drugs in Dutch Idiopathic Pulmonary Fibrosis Patients. Lung.

[13] Man R.K., Gogikar A., Nanda A., Janga L.S.N., Sambe H.G., Yasir M., Ramphall S. (2024). A Comparison of the Effectiveness of Nintedanib and Pirfenidone in Treating Idiopathic Pulmonary Fibrosis: A Systematic Review. Cureus.

[14] Chianese M., Screm G., Salton F., Confalonieri P., Trotta L., Barbieri M., Ruggero L., Mari M., Reccardini N., Geri P. (2024). Pirfenidone and Nintedanib in Pulmonary Fibrosis: Lights and Shadows. Pharmaceuticals.

[15] Laporta Hernandez R., Aguilar Perez M., Lázaro Carrasco M.T., Ussetti Gil P. (2018). Lung Transplantation in Idiopathic Pulmonary Fibrosis. Med. Sci..

[16] Mia M.M., Singh M.K. (2022). New Insights into Hippo/YAP Signaling in Fibrotic Diseases. Cells.

[17] Staley B.K., Irvine K.D. (2012). Hippo Signaling in Drosophila: Recent Advances and Insights. Dev. Dyn. Off. Publ. Am. Assoc. Anat..

[18] Liu H., Du S., Lei T., Wang H., He X., Tong R., Wang Y. (2018). Multifaceted Regulation and Functions of YAP/TAZ in Tumors (Review). Oncol. Rep..

[19] Ortega Á., Vera I., Diaz M., Navarro C., Rojas M., Torres W., Parra H., Salazar J., De Sanctis J., Bermúdez V. (2021). The YAP/TAZ Signaling Pathway in the Tumor Microenvironment and Carcinogenesis: Current Knowledge and Therapeutic Promises. Int. J. Mol. Sci..

[20] Papavassiliou K.A., Sofianidi A.A., Papavassiliou A.G. (2024). YAP/TAZ-TEAD Signalling Axis: A New Therapeutic Target in Malignant Pleural Mesothelioma. J. Cell. Mol. Med..

[21] Pocaterra A., Romani P., Dupont S. (2020). YAP/TAZ Functions and Their Regulation at a Glance. J. Cell Sci..

[22] McDonald C.B., McIntosh S.K.N., Mikles D.C., Bhat V., Deegan B.J., Seldeen K.L., Saeed A.M., Buffa L., Sudol M., Nawaz Z. (2011). Biophysical Analysis of Binding of WW Domains of the YAP2 Transcriptional Regulator to PPXY Motifs within WBP1 and WBP2 Adaptors. Biochemistry.

[23] Boopathy G.T.K., Hong W. (2019). Role of Hippo Pathway-YAP/TAZ Signaling in Angiogenesis. Front. Cell Dev. Biol..

[24] Fu M., Hu Y., Lan T., Guan K.-L., Luo T., Luo M. (2022). The Hippo Signalling Pathway and Its Implications in Human Health and Diseases. Signal Transduct. Target. Ther..

[25] Luo J., Zou H., Guo Y., Tong T., Chen Y., Xiao Y., Pan Y., Li P. (2023). The Oncogenic Roles and Clinical Implications of YAP/TAZ in Breast Cancer. Br. J. Cancer.

[26] Moon H., Park H., Chae M.J., Choi H.J., Kim D.Y., Ro S.W. (2022). Activated TAZ Induces Liver Cancer in Collaboration with EGFR/HER2 Signaling Pathways. BMC Cancer.

[27] Hao G., Lv T., Wu Y., Wang H., Xing W., Wang Y., Li C., Zhang Z., Wang Z., Wang W. (2017). The Hippo Signaling Pathway: A Potential Therapeutic Target Is Reversed by a Chinese Patent Drug in Rats with Diabetic Retinopathy. BMC Complement. Altern. Med..

[28] Xie J., Wang Y., Ai D., Yao L., Jiang H. (2022). The Role of the Hippo Pathway in Heart Disease. FEBS J..

[29] Bertero T., Oldham W.M., Cottrill K.A., Pisano S., Vanderpool R.R., Yu Q., Zhao J., Tai Y., Tang Y., Zhang Y.-Y. (2016). Vascular Stiffness Mechanoactivates YAP/TAZ-Dependent Glutaminolysis to Drive Pulmonary Hypertension. J. Clin. Investig..

[30] Qian X., He L., Hao M., Li Y., Li X., Liu Y., Jiang H., Xu L., Li C., Wu W. (2021). YAP Mediates the Interaction between the Hippo and PI3K/Akt Pathways in Mesangial Cell Proliferation in Diabetic Nephropathy. Acta Diabetol..

[31] Gogia N., Chimata A.V., Deshpande P., Singh A., Singh A. (2021). Hippo Signaling: Bridging the Gap between Cancer and Neurodegenerative Disorders. Neural Regen. Res..

[32] Hong L., Li X., Zhou D., Geng J., Chen L. (2018). Role of Hippo Signaling in Regulating Immunity. Cell. Mol. Immunol..

[33] Fu S., Zhao W., Zhang W., Song H., Ji H., Tang N. (2017). Hippo Signaling Pathway in Lung Development, Regeneration, and Diseases. Yi Chuan Hered..

[34] Xie H., Wu L., Deng Z., Huo Y., Cheng Y. (2018). Emerging Roles of YAP/TAZ in Lung Physiology and Diseases. Life Sci..

[35] Byrne A.J., Maher T.M., Lloyd C.M. (2016). Pulmonary Macrophages: A New Therapeutic Pathway in Fibrosing Lung Disease?. Trends Mol. Med..

[36] Liu F., Lagares D., Choi K.M., Stopfer L., Marinković A., Vrbanac V., Probst C.K., Hiemer S.E., Sisson T.H., Horowitz J.C. (2015). Mechanosignaling through YAP and TAZ Drives Fibroblast Activation and Fibrosis. Am. J. Physiol.-Lung Cell. Mol. Physiol..

[37] Piccolo S., Dupont S., Cordenonsi M. (2014). The Biology of YAP/TAZ: Hippo Signaling and Beyond. Physiol. Rev..

[38] Ghosh A.K., Vaughan D.E. (2012). PAI-1 in Tissue Fibrosis. J. Cell. Physiol..

[39] Samarakoon R., Higgins S.P., Higgins C.E., Higgins P.J. (2008). TGF-Beta1-Induced Plasminogen Activator Inhibitor-1 Expression in Vascular Smooth Muscle Cells Requires Pp60(c-Src)/EGFR(Y845) and Rho/ROCK Signaling. J. Mol. Cell. Cardiol..

[40] Link P.A., Choi K.M., Diaz Espinosa A.M., Jones D.L., Gao A.Y., Haak A.J., Tschumperlin D.J. (2022). Combined Control of the Fibroblast Contractile Program by YAP and TAZ. Am. J. Physiol.-Lung Cell. Mol. Physiol..

[41] Luo J., Yu F.-X. (2019). GPCR-Hippo Signaling in Cancer. Cells.

[42] Zmajkovicova K., Bauer Y., Menyhart K., Schnoebelen M., Freti D., Boucher M., Renault B., Studer R., Birker-Robaczewska M., Klenk A. (2020). GPCR-Induced YAP Activation Sensitizes Fibroblasts to Profibrotic Activity of TGFβ1. PLoS ONE.

[43] Milara J., Navarro R., Juan G., Peiró T., Serrano A., Ramón M., Morcillo E., Cortijo J. (2012). Sphingosine-1-Phosphate Is Increased in Patients with Idiopathic Pulmonary Fibrosis and Mediates Epithelial to Mesenchymal Transition. Thorax.

[44] Huang L.S., Sudhadevi T., Fu P., Punathil-Kannan P.-K., Ebenezer D.L., Ramchandran R., Putherickal V., Cheresh P., Zhou G., Ha A.W. (2020). Sphingosine Kinase 1/S1P Signaling Contributes to Pulmonary Fibrosis by Activating Hippo/YAP Pathway and Mitochondrial Reactive Oxygen Species in Lung Fibroblasts. Int. J. Mol. Sci..

[45] Huang L.S., Berdyshev E., Mathew B., Fu P., Gorshkova I.A., He D., Ma W., Noth I., Ma S.-F., Pendyala S. (2013). Targeting Sphingosine Kinase 1 Attenuates Bleomycin-Induced Pulmonary Fibrosis. FASEB J. Off. Publ. Fed. Am. Soc. Exp. Biol..

[46] Aravamudhan A., Haak A.J., Choi K.M., Meridew J.A., Caporarello N., Jones D.L., Tan Q., Ligresti G., Tschumperlin D.J. (2020). TBK1 Regulates YAP/TAZ and Fibrogenic Fibroblast Activation. Am. J. Physiol.-Lung Cell. Mol. Physiol..

[47] Helgason E., Phung Q.T., Dueber E.C. (2013). Recent Insights into the Complexity of Tank-binding Kinase 1 Signaling Networks: The Emerging Role of Cellular Localization in the Activation and Substrate Specificity of TBK1. FEBS Lett..

[48] Lin K.C., Moroishi T., Meng Z., Jeong H.-S., Plouffe S.W., Sekido Y., Han J., Park H.W., Guan K.-L. (2017). Regulation of Hippo Pathway Transcription Factor TEAD by P38 MAPK-Induced Cytoplasmic Translocation. Nat. Cell Biol..

[49] Chen Y., Zhao X., Sun J., Su W., Zhang L., Li Y., Liu Y., Zhang L., Lu Y., Shan H. (2019). YAP1/Twist Promotes Fibroblast Activation and Lung Fibrosis That Conferred by miR-15a Loss in IPF. Cell Death Differ..

[50] Lee K.-W., Yeo S.-Y., Sung C.O., Kim S.-H. (2015). Twist1 Is a Key Regulator of Cancer-Associated Fibroblasts. Cancer Res..

[51] Tan J., Tedrow J.R., Nouraie M., Dutta J.A., Miller D.T., Li X., Yu S., Chu Y., Juan-Guardela B., Kaminski N. (2017). Loss of Twist1 in the Mesenchymal Compartment Promotes Increased Fibrosis in Experimental Lung Injury by Enhanced Expression of CXCL12. J. Immunol..

[52] Zhu T., Ma Z., Wang H., Jia X., Wu Y., Fu L., Li Z., Zhang C., Yu G. (2020). YAP/TAZ Affects the Development of Pulmonary Fibrosis by Regulating Multiple Signaling Pathways. Mol. Cell. Biochem..

[53] Gokey J.J., Sridharan A., Xu Y., Green J., Carraro G., Stripp B.R., Perl A.-K.T., Whitsett J.A. (2018). Active Epithelial Hippo Signaling in Idiopathic Pulmonary Fibrosis. JCI Insight.

[54] Antoniou K.M., Margaritopoulos G.A., Soufla G., Symvoulakis E., Vassalou E., Lymbouridou R., Samara K.D., Kappou D., Spandidos D.A., Siafakas N.M. (2010). Expression Analysis of Akt and MAPK Signaling Pathways in Lung Tissue of Patients with Idiopathic Pulmonary Fibrosis (IPF). J. Recept. Signal Transduct..

[55] Sun Z., Gong X., Zhu H., Wang C., Xu X., Cui D., Qian W., Han X. (2014). Inhibition of Wnt/Β- C Atenin Signaling Promotes Engraftment of Mesenchymal Stem Cells to Repair Lung Injury. J. Cell. Physiol..

[56] Akhmetshina A., Palumbo K., Dees C., Bergmann C., Venalis P., Zerr P., Horn A., Kireva T., Beyer C., Zwerina J. (2012). Activation of Canonical Wnt Signalling Is Required for TGF-β-Mediated Fibrosis. Nat. Commun..

[57] Liu T., Hu B., Choi Y.Y., Chung M., Ullenbruch M., Yu H., Lowe J.B., Phan S.H. (2009). Notch1 Signaling in FIZZ1 Induction of Myofibroblast Differentiation. Am. J. Pathol..

[58] Yan P., Liu J., Li Z., Wang J., Zhu Z., Wang L., Yu G. (2023). Glycolysis Reprogramming in Idiopathic Pulmonary Fibrosis: Unveiling the Mystery of Lactate in the Lung. Int. J. Mol. Sci..

[59] Chung K.-P., Hsu C.-L., Fan L.-C., Huang Z., Bhatia D., Chen Y.-J., Hisata S., Cho S.J., Nakahira K., Imamura M. (2019). Mitofusins Regulate Lipid Metabolism to Mediate the Development of Lung Fibrosis. Nat. Commun..

[60] Koudelka A., Cechova V., Rojas M., Mitash N., Bondonese A., St. Croix C., Ross M.A., Freeman B.A. (2022). Fatty Acid Nitroalkene Reversal of Established Lung Fibrosis. Redox Biol..

[61] Li J., Zhai X., Sun X., Cao S., Yuan Q., Wang J. (2022). Metabolic Reprogramming of Pulmonary Fibrosis. Front. Pharmacol..

[62] Umeda Y., Demura Y., Morikawa M., Anzai M., Kadowaki M., Ameshima S., Tsuchida T., Tsujikawa T., Kiyono Y., Okazawa H. (2015). Prognostic Value of Dual-Time-Point 18F-FDG PET for Idiopathic Pulmonary Fibrosis. J. Nucl. Med. Off. Publ. Soc. Nucl. Med..

[63] Kashihara T., Mukai R., Oka S., Zhai P., Nakada Y., Yang Z., Mizushima W., Nakahara T., Warren J.S., Abdellatif M. (2022). YAP Mediates Compensatory Cardiac Hypertrophy through Aerobic Glycolysis in Response to Pressure Overload. J. Clin. Investig..

[64] Koo J.H., Guan K.-L. (2018). Interplay between YAP/TAZ and Metabolism. Cell Metab..

[65] Yin X., Choudhury M., Kang J.-H., Schaefbauer K.J., Jung M.-Y., Andrianifahanana M., Hernandez D.M., Leof E.B. (2019). Hexokinase 2 Couples Glycolysis with the Profibrotic Actions of TGF-β. Sci. Signal..

[66] Enzo E., Santinon G., Pocaterra A., Aragona M., Bresolin S., Forcato M., Grifoni D., Pession A., Zanconato F., Guzzo G. (2015). Aerobic Glycolysis Tunes YAP/TAZ Transcriptional Activity. EMBO J..

[67] Nguyen V., Taine E.G., Meng D., Cui T., Tan W. (2024). Pharmacological Activities, Therapeutic Effects, and Mechanistic Actions of Trigonelline. Int. J. Mol. Sci..

[68] Mohamadi N., Sharififar F., Pournamdari M., Ansari M. (2018). A Review on Biosynthesis, Analytical Techniques, and Pharmacological Activities of Trigonelline as a Plant Alkaloid. J. Diet. Suppl..

[69] Allred K.F., Yackley K.M., Vanamala J., Allred C.D. (2009). Trigonelline Is a Novel Phytoestrogen in Coffee Beans. J. Nutr..

[70] Ashihara H., Ludwig I.A., Katahira R., Yokota T., Fujimura T., Crozier A. (2015). Trigonelline and Related Nicotinic Acid Metabolites: Occurrence, Biosynthesis, Taxonomic Considerations, and Their Roles in Planta and in Human Health. Phytochem. Rev..

[71] Zeyada M.S., Eraky S.M., El-Shishtawy M.M. (2024). Trigonelline Mitigates Bleomycin-Induced Pulmonary Inflammation and Fibrosis: Insight into NLRP3 Inflammasome and SPHK1/S1P/Hippo Signaling Modulation. Life Sci..

[72] Haak A.J., Kostallari E., Sicard D., Ligresti G., Choi K.M., Caporarello N., Jones D.L., Tan Q., Meridew J., Diaz Espinosa A.M. (2019). Selective YAP/TAZ Inhibition in Fibroblasts via Dopamine Receptor D1 Agonism Reverses Fibrosis. Sci. Transl. Med..

[73] Moya I.M., Halder G. (2019). Hippo–YAP/TAZ Signalling in Organ Regeneration and Regenerative Medicine. Nat. Rev. Mol. Cell Biol..

[74] Kim J., Kim Y.H., Kim J., Park D.Y., Bae H., Lee D.-H., Kim K.H., Hong S.P., Jang S.P., Kubota Y. (2017). YAP/TAZ Regulates Sprouting Angiogenesis and Vascular Barrier Maturation. J. Clin. Investig..

[75] Santos D.M., Pantano L., Pronzati G., Grasberger P., Probst C.K., Black K.E., Spinney J.J., Hariri L.P., Nichols R., Lin Y. (2020). Screening for YAP Inhibitors Identifies Statins as Modulators of Fibrosis. Am. J. Respir. Cell Mol. Biol..

[76] Zhao X., Sun J., Su W., Shan H., Zhang B., Wang Y., Shabanova A., Shan H., Liang H. (2018). Melatonin Protects against Lung Fibrosis by Regulating the Hippo/YAP Pathway. Int. J. Mol. Sci..

[77] Newman D.J., Cragg G.M. (2020). Natural Products as Sources of New Drugs over the Nearly Four Decades from 01/1981 to 09/2019. J. Nat. Prod..

[78] Li C., Li Q., Mei Q., Lu T. (2015). Pharmacological Effects and Pharmacokinetic Properties of Icariin, the Major Bioactive Component in Herba Epimedii. Life Sci..

[79] Huang H., Zhang Z., Qin F., Tang W., Liu D., Wu P., Jiao F. (2019). Icariin Inhibits Chondrocyte Apoptosis and Angiogenesis by Regulating the TDP-43 Signaling Pathway. Mol. Genet. Genomic Med..

[80] Singh W.R., Devi H.S., Kumawat S., Sadam A., Appukuttan A.V., Patel M.R., Lingaraju M.C., Singh T.U., Kumar D. (2019). Angiogenic and MMPs Modulatory Effects of Icariin Improved Cutaneous Wound Healing in Rats. Eur. J. Pharmacol..

[81] Li Y., Ding X., Li H., Zhang C. (2013). Icariin Attenuates High Glucose-induced Type IV Collagen and Fibronectin Accumulation in Glomerular Mesangial Cells by Inhibiting Transforming Growth Factor-β Production and Signalling through G Protein-coupled Oestrogen Receptor 1. Clin. Exp. Pharmacol. Physiol..

[82] Sun X., Cheng H., Liu B., Du Y., Dong J., Huang J. (2020). Icariin Reduces LPS-Induced Acute Lung Injury in Mice Undergoing Bilateral Adrenalectomy by Regulating GRα. Eur. J. Pharmacol..

[83] Du W., Tang Z., Yang F., Liu X., Dong J. (2021). Icariin Attenuates Bleomycin-Induced Pulmonary Fibrosis by Targeting Hippo/YAP Pathway. Biomed. Pharmacother..

[84] Zmajkovicova K., Menyhart K., Bauer Y., Studer R., Renault B., Schnoebelen M., Bolinger M., Nayler O., Gatfield J. (2019). The Antifibrotic Activity of Prostacyclin Receptor Agonism Is Mediated through Inhibition of YAP/TAZ. Am. J. Respir. Cell Mol. Biol..

[85] Paul S.M., Mytelka D.S., Dunwiddie C.T., Persinger C.C., Munos B.H., Lindborg S.R., Schacht A.L. (2010). How to Improve R&D Productivity: The Pharmaceutical Industry’s Grand Challenge. Nat. Rev. Drug Discov..

[86] Li M., Zhou Y., Wang T., Li M., Chen X., Zhang T., Wang D., Zhang J. (2022). Nintedanib Exerts Anti-Pulmonary Fibrosis Activity via Inhibiting TANK-Binding Kinase 1 (TBK1) Phosphorylation. Chem. Commun..

[87] Graham K., Lienau P., Bader B., Prechtl S., Naujoks J., Lesche R., Weiske J., Kuehnlenz J., Brzezinka K., Potze L. (2024). Discovery of YAP1/TAZ Pathway Inhibitors through Phenotypic Screening with Potent Anti-Tumor Activity via Blockade of Rho-GTPase Signaling. Cell Chem. Biol..

[88] Huang L., Yang X., Feng Y., Huang H.-X., Hu J.-Q., Yan P.-Y., Pan H.-D., Xie Y. (2024). ShaShen-MaiDong Decoction Attenuates Bleomycin-Induced Pulmonary Fibrosis by Inhibiting TGF-β/Smad3, AKT/MAPK, and YAP/TAZ Pathways. J. Ethnopharmacol..

[89] Ohto-Fujita E., Shimizu M., Atomi A., Hiruta H., Hosoda R., Horinouchi S., Miyazaki S., Murakami T., Asano Y., Hasebe Y. (2024). Eggshell Membrane and Its Major Component Lysozyme and Ovotransferrin Enhance the Secretion of Decorin as an Endogenous Antifibrotic Mediator from Lung Fibroblasts and Ameliorate Bleomycin-Induced Pulmonary Fibrosis. Biochem. Biophys. Rep..

